# Neural Associations of the Early Retinotopic Cortex with the Lateral Occipital Complex during Visual Perception

**DOI:** 10.1371/journal.pone.0108557

**Published:** 2014-09-24

**Authors:** Delong Zhang, Xue Wen, Bishan Liang, Bo Liu, Ming Liu, Ruiwang Huang

**Affiliations:** 1 Department of Radiology, Guangdong Provincial Hospital of Chinese Medicine, Guangzhou, China; 2 Center for the Study of Applied Psychology, Key Laboratory of Mental Health and Cognitive Science of Guangdong Province, School of Psychology, South China Normal University, Guangzhou, China; 3 Guangzhou University of Chinese Medicine postdoctoral mobile research station, Guangzhou, China; Monash University, Australia

## Abstract

Previous studies have demonstrated that the early retinotopic cortex (ERC, i.e., V1/V2/V3) is highly associated with the lateral occipital complex (LOC) during visual perception. However, it remains largely unclear how to evaluate their associations in quantitative way. The present study tried to apply a multivariate pattern analysis (MVPA) to quantify the neural activity in ERC and its association with that of the LOC when participants saw visual images. To this end, we assessed whether low-level visual features (Gabor features) could predict the neural activity in the ERC and LOC according to a voxel-based encoding model (VBEM), and then quantified the association of the neural activity between these regions by using an analogical VBEM. We found that the Gabor features remarkably predicted the activity of the ERC (e.g., the predicted accuracy was 52.5% for a participant) instead of that of the LOC (4.2%). Moreover, the MVPA approach can also be used to establish corresponding relationships between the activity patterns in the LOC and those in the ERC (64.2%). In particular, we found that the integration of the Gabor features and LOC visual information could dramatically improve the ‘prediction’ of ERC activity (88.3%). Overall, the present study provides new evidences for the possibility of quantifying the association of the neural activity between the regions of ERC and LOC. This approach will help to provide further insights into the neural substrates of the visual processing.

## Introduction

The early retinotopic cortex (ERC) constitutes of the primary visual cortex (V1) and extrastriate visual cortical areas (e.g., V2, V3) and is responsible for processing visual information. The neural activity in the ERC is observed to be highly associated with the lateral occipital complex (LOC) during visual perception [1]–[3] and these regions jointly comprises a hierarchical axis for representation of visual properties in the ventral visual pathway [4]. However, few studies have directly evaluated the association of the neural activity across these regions in quantitative way during visual processing.

The neural activity of the ERC complexly interacted with that of the LOC during visual processing. Previous studies demonstrated that the visual cortex contributed differently to the object neural representation [5], [6]. Particularly, the neural activity in the ERC veridically represents the low-level properties of the visual image, and then the neural representation as an input is further translated to the high-level cortical regions (e.g., LOC) to acquire its meanings. Through manipulating the properties of the outside stimuli in experiments, many studies demonstrated that the neural activity in the ERC was responsible for the local properties of the outside stimuli but that in the LOC for the perceived stimuli [7]–[10]. However, recent findings suggest that the ERC is not a static spatiotemporal filter for local features; rather, it reflects the interpretations and meanings of the processed stimuli [11]. By directly manipulating the high-level interpretation of the stimulus, several studies have provided additional evidences for understanding the neural interaction of the ERC and LOC in object representation and suggested that the neural activity in the ERC reflects the retinal image as well as the perceived stimuli features during the visual processing [1], [12], [13]. However, we noted that this knowledge was primarily derived from the performance of neural activity in these regions based on different experiment designs. It is consequently warrant to directly measuring the relationship of the neural activities of these regions during the visual processing.

Recently, the voxel-based encoding model (VBEM), a method of multivariate pattern analysis (MVPA), has been proposed [14]–[17] to quantitatively explore whether some specific features (e.g., visual/semantic features) are represented in the activity of an individual voxel (for review, see [16]). For the first time, Kay et al. [14] trained a VBEM to identify the relationships between the Gabor features (including three low-level visual dimensions, i.e., spatial position, spatial frequency, and orientation) of visual stimuli and each voxel's activity in the ERC. As such, they built linear mappings between the visual stimuli and related ERC activities [14]. Similarly, Mitchell et al. observed mapping between brain activity and semantic patterns of concrete nouns (i.e., frequency of text co-occurrence with appointed verbs) by using a VBEM [15]. These findings imply that a VBEM can be used to precisely identify the neural representations in brain activities in visual cortex and therefore provides an opportunity to quantify the association between neural activities in the ERC and LOC.

In the current study, we applied a VBEM method to evaluate the neural representation of the ERC by the low-level properties of the visual object, and an analogical approach (i.e., analogical voxel-based encoding method, AVBEM) for the first time to depict the quantitative association of the neural activities between the ERC and LOC when participants were shown images of natural settings. First, we examined whether a VBEM could capture the linear mappings between Gabor feature patterns and neural activity in the ERC. Then, we tested whether linear mappings also exist between neural activity in the LOC and that in the ERC by using an AVBEM. Finally, we evaluated the combination of VBEM and AVBEM by integrating Gabor features of stimuli and LOC visual information to ‘predict’ brain activity in the ERC.

## Method and Materials

### Data Acquisition

The visual experimental fMRI datasets were provided by the Gallant's lab in University of California, Berkeley, and publicly available at the website of Dr. Gallant's lab (http://crcns.org). The University of California, Berkeley Committee for the Protection of Human Subjects approved all the experimental protocol. These fMRI datasets include the estimated BOLD data and the experimental stimuli, which were used to identify natural images from brain activity using an encoding model in two previous studies [14], [18]. The experimental design, stimuli, fMRI parameters, and data preprocessing procedure have been described in these previous studies [14], [18]. In brief, functional data were collected from two participants who were instructed to view natural images during BOLD-fMRI scanning. For each participant, the total experiment was divided into five sessions to avoid fatigue. Each session consisted of five runs for model estimation and two runs for model validation. Each run for model estimation lasted 11 minutes and consisted of 70 distinct natural images presented twice each, whereas each run used for model validation lasted 12 minutes and consisted of 12 distinct natural images presented 13 times each [14]. In each trial within each run, a stimulus (grayscale natural images) was flashed at 200-ms intervals (On-Off-On-Off-On) for 1 s, followed by 3 s of gray background. In total, 1750 training images were collected for model estimation for each participant, and 120 testing images were obtained for model validation. All MRI data were scanned at the Brain Imaging Center at UC Berkeley using a 4 T INOVA MRI scanner (Varian, Inc.) with a quadrature transmit/receive surface coil (Midwest RF, LLC). The BOLD-fMRI data were acquired in 18 coronal slices that covered the occipital cortex with a gradient-echo EPI pulse sequence (TR  = 1 s, TE  = 28 ms, flip angle  = 20°, matrix size  = 64×64, FOV  = 128×128 mm^2^, and slice thickness/gap  = 2.25/0.25 mm).

### Analysis Framework

**Figure 1:**
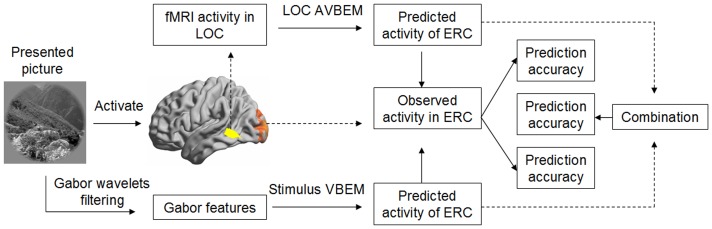
Overview of the data analysis framework.

Fig. 1 illustrates the data analysis framework of the current study. First, we selected two regions of interest (ROIs), ERC and LOC, according to a previous study [14]. Then, we extracted the fMRI activity patterns of each natural image in the ERC and LOC for each participant. In the model training stage, we adopted both VBEM and AVBEM to describe the relationships between neural activity in the ERC and the visual features of the stimulus, and activity pattern in the LOC, respectively. In total, we used three types of approaches: the stimulus VBEM (Gabor feature pattern), LOC AVBEM (LOC pattern), and combination of VBEM and AVBEM (LOC pattern and Gabor feature pattern). Finally, we applied the testing images to validate the trained VBEM, AVBEM, and their combinations. A step-by-step description of the detailed procedure is as follows:

### Data preprocessing

#### Preprocessing of Natural Images

We preprocessed natural image similar to the approaches used in previous studies [14], [18]. First, all natural images were filtered by a set of Gabor wavelet. In detail, the Gabor wavelet was defined as a spatially localized filter with a specific orientation and spatial frequency. The Gabor wavelets occurred at six spatial frequencies: 1, 2, 4, 8, 16, and 32 cycles/FOV (FOV  = 20° covering a downsampled resolution of 128×128 pixels in this analysis). At each spatial frequency of *n* cycles/FOV, wavelets were located on an *n*×*n* grid that tiled the full FOV. At each grid position, wavelets occurred at eight orientations: 0°, 22.5°, 45°, 67.5°, 90°, 112.5°, 135°, and 157.5°. A luminance-only wavelet, which covered the entire natural image, was also included. We thus produced all of the Gabor wavelets (total number  =  (1+22+42+82+162+322) ×8+1 = 10921). All of the calculations were performed in Matlab. Then, we performed a nonlinear transformation on each filtered natural image by using the log of the magnitude, as the log function could improve the prediction accuracy with a compressive nonlinearity [18]. Finally, we obtained a feature vector (10921×1) for each natural image and used it as the input channel (details are shown below) for the stimulus encoding model.

#### Extraction of the Brain Activity Pattern

Based on the BOLD-fMRI signals, we extracted the fMRI activity patterns of the ERC and LOC for 1750 training and 120 testing natural images separately. The fMRI signal patterns in ERC were used as the output channels (details are shown below) for the three encoding ‘models’ (i.e., VBEM, AVBEM, and the combination of VBEM and AVBEM), and the LOC patterns were used as the input channel (details are shown below) for the LOC AVBEM.

### Stimulus VBEM

The construction of the stimulus encoding model included two stages: one was the model estimation to establish the relationships between the Gabor features of the training natural images and the corresponding fMRI activity patterns in the ERC; and the other was the model validation to assess the performance of the trained model using the tested natural images to predict the activity patterns in the ERC.

#### Model Estimation

**Figure 2:**
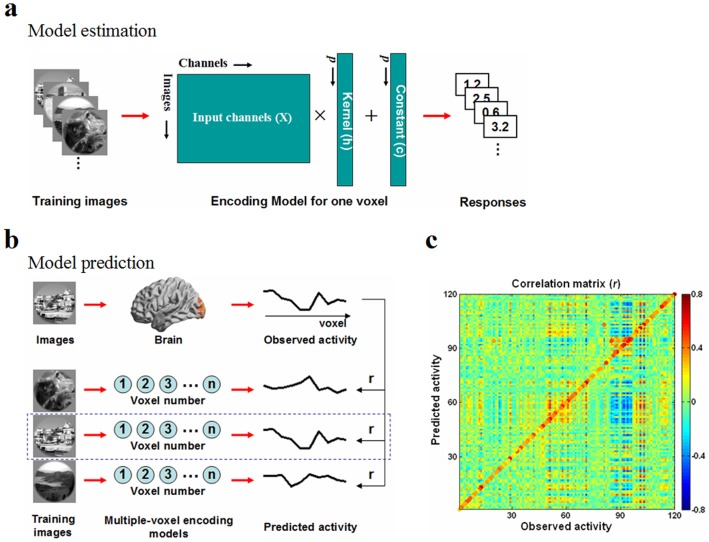
Schematic of the voxel-based encoding model used in this study. (a) Model estimation. We used training images as input channels to estimate the encoding model of one voxel with a gradient descent algorithm. (b) Model prediction. The correlation *r* was calculated between the observed activity pattern (i.e., the fMRI response to each training image in the early visual cortex) and the predicted activity pattern from multiple encoding models. The dotted box represents the most closely matched model. (c) Correlation matrix and prediction performance. The color at the (*n, m*) element represents the correlation between the observed activity pattern for the *m*th image and the predicted activity pattern for the *n*th image. The maximum correlation in each column is designated by an enlarged circle of the appropriate color, which indicates the image selected by the prediction algorithm. If the diagonal element was the maximum value in each column, we marked it as a correct prediction. The prediction performance of the encoding model was defined as the ratio of the number of correct predictions to the total number of training images. For this participant, the performance was 88.3% (106/120).

As shown in Fig. 2a, we established the stimulus encoding model in a multiple voxel-based manner [14] to analyze the fMRI signal of each voxel in ERC. We let *p* be the number of training natural images and *q* be the number of training images or input channels. For a voxel in the ERC, we assumed that its response can be expressed by using a general linear model (GLM),
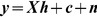
(1)where ***y*** is the set of output channels (i.e., the responses of all voxels in ERC) with the dimension of (*p*×1), ***X*** is the set of input channels (*p*×*q*), ***h*** is the kernel (*q*×1), ***c*** is the constant (*p*×1), and ***n*** is the noise (*p*×1). In the calculations, we used the functions in the STRFlab toolbox (Version 1.45, http://strflab.berkeley.edu/) to automatically estimate the model parameters. In detail, we adopted a single voxel stimulus encoding model or eq.(1), set the input channels to the Gabor features used in training with natural images (1750×10921), and obtained output channels representing the fMRI signal of a voxel in ERC (1750×1). Model parameters were estimated using a gradient descent with an early stopping approach [14], [19], in which the magnitude of model parameter estimates were shrunk to prevent over-fitting and 20% of the responses were randomly selected to comprise the stopping set. A bootstrap sampling approach was used for iterative analysis.

The above procedures were repeated across all voxels in the ERC. Finally, we obtained a multiple-voxel model as the stimulus encoding model. Thus, we were able to predict the brain fMRI activity pattern in the ERC while the participants viewed natural images.

#### Model Validation

To validate the model performance, we applied the 120 testing natural images that were not contained in the model estimation stage to build the activity pattern in the ERC (Fig. 2b). For each of the 120 testing images, the fMRI responses to the image in the ERC were defined as the ‘observed activity pattern’. Thus, we had a total of 120 observed activity patterns for model validation. The Gabor features of each testing image were entered into the stimulus encoding model to calculate the ‘predicted activity pattern’ of the image according to eq. (1). In this manner, we obtained 120 predicted activity patterns in the ERC. Then, we calculated the Pearson′s correlation coefficient between each pair of the two sets of patterns and obtained a 120-by-120 correlation matrix for each participant (Fig. 2c). If the diagonal element of the correlation matrix was the maximum value in each column, which means that the correlation between the predicted activity pattern of an image and the observed activity pattern of that image corresponds to the best match, we marked it as a correct prediction. Finally, the predictive performance of the stimulus encoding model was defined as the ratio of the number of the correct predictions to the total number of training images (corrects/120).

### LOC AVBEM

The procedures for constructing the LOC-related encoding method were similar to those used for the stimulus encoding model, except for the input channels. Based on the indices of voxels in the downloaded dataset, we determined that the number of voxels in the LOC was 928 for participant S1 and 358 for participant S2, respectively. By setting the input channels to represent the activity patterns of all voxels in the LOC (1750×928 for S1 and 1750×358 for S2) and the output channel to represent the fMRI activity patterns of all voxels in the ERC, we performed a ‘model’ estimation and ‘model’ validation analyses according to eq.(1).

### Combination of VBEM and AVBEM

We further performed linear combination of the stimulus Gabor feature and the LOC pattern information in term of a combination of VBEM and AVBEM. For each of the 120 testing images, we averaged the two predicted activity patterns obtained from the stimulus VBEM and the LOC AVBEM for the corresponding voxel and set the result as the predicted activity pattern of the ERC. The performance of the combined VBEM and AVBEM was then estimated by using a procedure similar to that described for the stimulus encoding model.

### Validation Analysis

In the present study, we further examined whether low-level visual features (i.e., Gabor features) could predict neural activity in the LOC using a stimulus encoding model for each participant.

## Results

**Figure 3:**
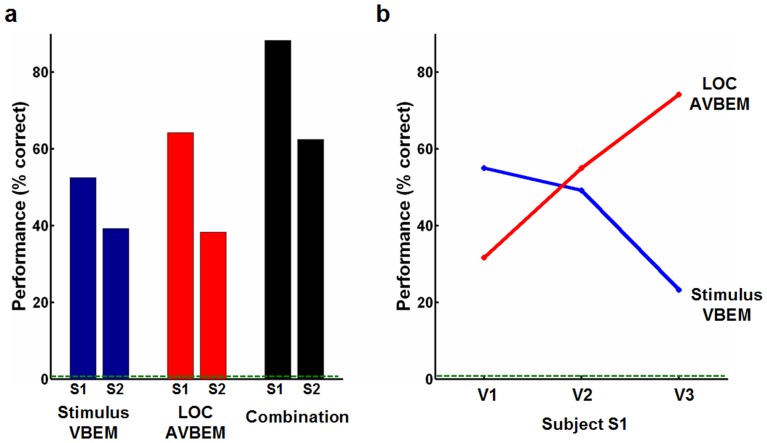
Performance of different voxel-based encoding models. (a) Summary of identification performance. The bars indicate the performance obtained from a set of 120 images, and the dashed green line indicates chance performance. Note that for both participants S1 and S2, the performance of all three methods was higher than the chance level, and the combination had higher performance than either the stimulus VBEM or the LOC AVBEM alone. (b) There were inverse modulations in the sub-areas of ERC between the stimulus VBEM and the LOC AVBEM. VBEM, voxel-based encoding model; AVBEM, analogical voxel-bsed encoding method.

We found that the MVPA approach could effectively predict the neural activity in the ERC (Fig. 3a). For the stimulus VBEM, the accuracy was 52.5% (63/120) for participant S1 and 39.2% (47/120) for participant S2, respectively. Notably, all of the predictive accuracies were remarkably higher than the chance performance of 0.8% (1/120). By contrast, there were no linear relationships between the Gabor feature patterns and the neural activity in the LOC. The accuracy of the VBEM for neural activity prediction in the LOC was only 4.2% for participant S1 and 0% for participant S2, respectively. For the LOC AVBEM, the accuracy was 64.2% (77/120) for participant S1 and 38.3% (46/120) for participant S2, respectively, which were also above the chance probability of 0.8% (1/120). For the combined VBEM and AVBEM, the accuracy was 88.3% (106/120) for participant S1 and 62.5% (75/120) for participant S2 (Fig. 3a). These accuracy values were higher than those of either the stimulus VBEM or the LOC AVBEM alone (Fig. 3a).

The contributions of the stimulus VBEM and the LOC AVBEM to neural activity prediction differed in the sub-regions of ERC (i.e., V1, V2, and V3). For participant S1, Fig. 3b shows that the prediction accuracy of the stimulus VBEM was 55.0% for V1, 49.2% for V2, and 23.3% for V3. In contrast, the accuracy derived from the LOC AVBEM was 31.7% for V1, 55.0% for V2, 74.2% for V3 (Fig. 3b). The dominance of neural activity prediction from visual properties ranged from V1 – V3, which is inverse to the ‘prediction’ from the LOC. This observation was validated by the results obtained from participant S2 (Fig. S1 in Text S1).

## Discussion

This study applied a voxel-based encoding method to quantify the neural activity of the ERC and its relationship with the neural activity of the LOC for visual processing. The main findings can be summarized as follows: (i) Gabor features were predictive of ERC activity when a linear relationship was constructed, but no such relationship existed between the Gabor features and LOC activity; (ii) an analogical encoding method could capture the linear relationship between neural activity in the LOC and that in the ERC, and (iii) a combination of the Gabor features and the LOC information could improve neural activity ‘prediction’ in the ERC.

The primary advance of this study may be that we further assessed the relationship of ERC activity and the high-level visual cortex (e.g., the LOC), which was not performed in a previous study [14]. The previous study demonstrated that the Gabor feature patterns could linearly describe voxel-based brain activity changes in the ERC [14], which provided evidence for the representation of low-level visual features in the ERC. The neural representation of the ERC will be further translated to the high-level visual cortex in the ventral visual pathway; the neural activity of the ERC is consequently associated with that of the LOC. Meanwhile, it is noteworthy that many studies have shown that ERC activity reflects high-level stimulus information such as perceived size [2], [20], grouping [2], [21], [22], brightness [23], and stimulus reward information [24]. Thus, neural activity in the ERC is not simply driven by low-level visual features; it is also affected by high-level visual cortical information [2], [3]. The investigation of the interaction relationship between the neural activities in the ERC and LOC will advance our understanding of the neural substrate for visual processing in the ventral visual pathway. By using a VBEM, once we had succeeded in building linear relationships between ERC activity and the Gabor features of the stimuli, we can construct similar relationships between the neural activity in the ERC and the high-level visual cortex information.

These object representations in the high-level visual cortex (e.g., the LOC) and in the ERC were distinct but corresponded to each other. Extant evidence has indicated that the LOC represents perceived object shape rather than low-level image features [10], [25]. Many studies have shown that shape representations in the LOC are independent of sensory modalities [26]–[29], but are highly influenced by the subjective experiences of the participants [30]. Consistent with these previous findings, the current study found that the neural activity in the ERC, but not in the LOC, could be predicted from the Gabor feature patterns of visual stimuli. Regardless, the neural activity in both the LOC and ERC may represent distinct levels of information of the same visual stimuli, as the brain activity in both the LOC and ERC contributed to the representation of the same object. For instance, Naselaris et al. [18] found that the semantic features of the stimuli could effectively predict the neural activity of the LOC in an encoding model. However, it is still an open issue whether the representations in the LOC comprised shape or semantic information during perception [31]. Nevertheless, corresponding relationships were still present between the neural representations in the LOC and those in the ERC, which may be simply derived from the visual input from the ERC to the LOC during visual processing, or the modulation effect of the LOC on the ERC, or both. Thus the nature of the complex relationship between them should be further studied in future.

We found that the linear addition of LOC information and Gabor features can improve the ‘prediction’ accuracy. A possible explanation is that the neural activity in the ERC represents a combination of purely physical stimulus properties and high-level visual input signals. Indeed, accumulating evidence shows that the neural activity in the ERC is heavily influenced by feedback from higher-level visual regions [11], [32]–[34], even when there is no bottom-up input [11]. Within the high-level visual cortex, the LOC is thought to be a critical region involved in this top-down modulation process [2], [3]. Previous studies of illusory contour processing explored the contributions of the early visual cortex and high-level visual cortex and clearly demonstrated that contour processing was initially observed in the LOC rather than in early visual regions [35]–[37]. These findings might suggest that neural activity in the ERC reflects feedback from higher-level regions (e.g., the LOC) [5] and are further supported by a recent study indicating that object recognition alters the fMRI spatial pattern in the ERC [1]. It is noteworthy that our findings in the present study can not be merely interpreted as the feedback of the LOC to the ERC during the visual processing, although the improved effect was observed by combining the VBEM and AVBEM.

The exact neural relationships between LOC and ERC need be further explored during visual perception. As we know, a hypothesis of Bayesian inference has been proposed within the visual system [38], [39]. According to this hypothesis, the visual areas calculate a likely inference for the processed stimuli in a hierarchical structure by integrating prior visual information from top-down signals with new bottom-up input [1]. Our findings might provide evidence for the integration of external input information and a top-down feedback signal from the high-level visual cortex in each sub-region (i.e., V1, V2 and V3) of ERC activity. Although this ‘modulation’ was obvious for ERC activity, we noted that the effects were different among sub-regions of the ERC. Specifically, the ‘modulation’ from the LOC was most significant in V3, then V2 and V1, which is the inverse of the influence of the Gabor features in the ERC.

It should be noted that the signal with non-neural sources (such as physiological (cardiac, respiratory) and motion noise [40]) contained in the fMRI data may complicate the interpretation to our findings. Although the fMRI data used in the present study had been preprocessed to reduce the autoregressive noise-related (i.e., physiological and motion noise) effects [14], the residual artifacts still cannot be removed completely [41], [42]. Several previous studies have demonstrated that the complete removal of noise from fMRI data is impossible nowadays [40], [41], [43]. Both the physiological and motion noises can influence the changes of brain signal and may even bias the correlation of fMRI signals between regions [43]. That is, these effects may affect the accuracy of using the signal in LOC to ‘predict’ that in ERC in the present study.

In order to validate our main findings, we also acquired the resting-state fMRI data from two subjects and estimated the neural association of the spontaneous activities between LOC and ERC by using the identical AVBEM analysis. We found that the neural association between ERC and LOC cannot be built on the basis of the resting-state fMRI data, and the influence of motion and global signals on the neural association was not significant (details see Text S1). These observations suggested that our findings may be mainly induced by the visual stimuli processing, neither by the motion noise nor by the spontaneous fluctuation of the brain in resting-state. In the future, the nature of the neural association between LOC and ERC during visual perception should be further explored by considering the physiological and motion effect.

An interesting extension of the current study is it may enable the visualization of mental imagery and its associated brain activity. The current study demonstrated that neural activity in the ERC exhibited linear relationships with both the Gabor feature and the neural patterns in the LOC. Therefore, we may indirectly build a corresponding relationship between LOC patterns and Gabor features. Many previous studies have shown that the neural pattern of visual objects is similar to that of visual imagery in the LOC [44]–[46]. We speculate that the “physical shell” of the mental imagery might be further reconstructed from brain activity on the basis of previous studies [18], which is one of the reasons that we selected the LOC from among the potential candidates in high-level visual cortex to be the target region to explore for top-down modulation effects in the ERC.

Several issues require further investigation in future studies. First, the current study focused on the LOC to detect the association of ERC activity and the high-level visual cortex. It would be interesting to investigate the other high-level visual cortical regions such as V3a, V3b, and V4 in this process [11]. Second, the performance of our stimulus encoding model was lower than that in previous studies [14]. The factors responsible for this difference should be considered in future studies. Third, the LOC was studied as a whole. However, many studies have shown sub-cortical regions exist within the LOC [47]–[49]; thus, future studies should further detect the different roles of the sub-cortical regions of LOC in the observed findings of the present study. The last but not the least, this study showed that the AVBEM approach made it possible to evaluate the covary of the LOC and ERC on the neural representation, however, the present study did not provide direct evidence for the modulation effect of the LOC on the neural activity of ERC. Thus, it is an interesting topic to further investigate their cause relationships by a fine experiment design.

## Conclusion

In summary, using VBEM and AVBEM methods, we evaluated the neural activity in the ERC and its association with the neural activity in the LOC when participants performed a simple visual task. We extended previous findings to show that representations of Gabor features in the ERC activity were relevance with the high-level visual cortex information (e.g., that in the LOC). And an AVBEM could evaluate the association of the neural activity between the ERC and the high-level visual cortex in quantitative way. This might provide new insights into the neural substrates underlying visual processing in the ventral visual pathway.

## Supporting Information

Text S1 **Supplementary Materials.**
(DOC)

## References

[1] Hsieh PJ, Vul E, Kanwisher N (2010) Recognition alters the spatial pattern of FMRI activation in early retinotopic cortex. J Neurophysiol 103: 1501–1507. 20071627 10.1152/jn.00812.2009 PMC3257064

[2] Murray SO, Kersten D, Olshausen BA, Schrater P, Woods DL (2002) Shape perception reduces activity in human primary visual cortex. Proc Natl Acad Sci U S A 99: 15164–15169. 12417754 10.1073/pnas.192579399 PMC137561

[3] Williams MA, Baker CI, Op de Beeck HP, Shim WM, Dang S, et al (2008) Feedback of visual object information to foveal retinotopic cortex. Nat Neurosci 11: 1439–1445. 18978780 10.1038/nn.2218 PMC2789292

[4] Lerner Y, Hendler T, Ben-Bashat D, Harel M, Malach R (2001) A hierarchical axis of object processing stages in the human visual cortex. Cerebral Cortex 11: 287–297. 11278192 10.1093/cercor/11.4.287

[5] Shpaner M, Molholm S, Forde E, Foxe JJ (2013) Disambiguating the roles of area V1 and the lateral occipital complex (LOC) in contour integration. Neuroimage 69: 146–156. 23201366 10.1016/j.neuroimage.2012.11.023 PMC3872825

[6] McKyton A, Zohary E (2007) Beyond retinotopic mapping: the spatial representation of objects in the human lateral occipital complex. Cerebral Cortex 17: 1164–1172. 16818474 10.1093/cercor/bhl027

[7] Kourtzi Z, Huberle E (2005) Spatiotemporal characteristics of form analysis in the human visual cortex revealed by rapid event-related fMRI adaptation. Neuroimage 28: 440–452. 16046147 10.1016/j.neuroimage.2005.06.017

[8] Yin C, Shimojo S, Moore C, Engel SA (2002) Dynamic shape integration in extrastriate cortex. Curr Biol 12: 1379–1385. 12194818 10.1016/s0960-9822(02)01071-0

[9] Lerner Y, Hendler T, Malach R (2002) Object-completion effects in the human lateral occipital complex. Cereb Cortex 12: 163–177. 11739264 10.1093/cercor/12.2.163

[10] Kourtzi Z, Kanwisher N (2001) Representation of perceived object shape by the human lateral occipital complex. Science 293: 1506–1509. 11520991 10.1126/science.1061133

[11] Bannert MM, Bartels A (2013) Decoding the yellow of a gray banana. Curr Biol 23: 2268–2272. 24184103 10.1016/j.cub.2013.09.016

[12] Sperandio I, Chouinard PA, Goodale MA (2012) Retinotopic activity in V1 reflects the perceived and not the retinal size of an afterimage. Nat Neurosci 15: 540–542. 22406550 10.1038/nn.3069

[13] Grill-Spector K, Kourtzi Z, Kanwisher N (2001) The lateral occipital complex and its role in object recognition. Vision Res 41: 1409–1422. 11322983 10.1016/s0042-6989(01)00073-6

[14] Kay KN, Naselaris T, Prenger RJ, Gallant JL (2008) Identifying natural images from human brain activity. Nature 452: 352–355. 18322462 10.1038/nature06713 PMC3556484

[15] Mitchell TM, Shinkareva SV, Carlson A, Chang KM, Malave VL, et al (2008) Predicting human brain activity associated with the meanings of nouns. Science 320: 1191–1195. 18511683 10.1126/science.1152876

[16] Naselaris T, Kay KN, Nishimoto S, Gallant JL (2011) Encoding and decoding in fMRI. Neuroimage 56: 400–410. 20691790 10.1016/j.neuroimage.2010.07.073 PMC3037423

[17] Schonwiesner M, Zatorre RJ (2009) Spectro-temporal modulation transfer function of single voxels in the human auditory cortex measured with high-resolution fMRI. Proc Natl Acad Sci U S A 106: 14611–14616. 19667199 10.1073/pnas.0907682106 PMC2732853

[18] Naselaris T, Prenger RJ, Kay KN, Oliver M, Gallant JL (2009) Bayesian reconstruction of natural images from human brain activity. Neuron 63: 902–915. 19778517 10.1016/j.neuron.2009.09.006 PMC5553889

[19] Tugnait JK (1994) Estimation of linear parametric models of nonGaussian discrete random fields with application to texture synthesis. IEEE Trans Image Process 3: 109–127. 18291913 10.1109/83.277894

[20] Fang F, Kersten D, Murray SO (2008) Perceptual grouping and inverse fMRI activity patterns in human visual cortex. J Vis 8: 2 1–9. 10.1167/8.7.2 19146235

[21] Fang F, Boyaci H, Kersten D, Murray SO (2008) Attention-dependent representation of a size illusion in human V1. Curr Biol 18: 1707–1712. 18993076 10.1016/j.cub.2008.09.025 PMC2638992

[22] Murray SO, Boyaci H, Kersten D (2006) The representation of perceived angular size in human primary visual cortex. Nat Neurosci 9: 429–434. 16462737 10.1038/nn1641

[23] Boyaci H, Fang F, Murray SO, Kersten D (2007) Responses to lightness variations in early human visual cortex. Curr Biol 17: 989–993. 17540572 10.1016/j.cub.2007.05.005 PMC1931490

[24] Serences JT (2008) Value-based modulations in human visual cortex. Neuron 60: 1169–1181. 19109919 10.1016/j.neuron.2008.10.051 PMC3384552

[25] Kourtzi Z (2001) Processing of perceived visual shape in the human lateral occipital complex. Perception 30: 11–11.

[26] Grill-Spector K, Kourtzi Z, Kanwisher N (2001) The lateral occipital complex and its role in object recognition. Vision Research 41: 1409–1422. 11322983 10.1016/s0042-6989(01)00073-6

[27] Amedi A, Jacobson G, Hendler T, Malach R, Zohary E (2002) Convergence of visual and tactile shape processing in the human lateral occipital complex. Cerebral Cortex 12: 1202–1212. 12379608 10.1093/cercor/12.11.1202

[28] Beauchamp MS (2005) See me, hear me, touch me: multisensory integration in lateral occipital-temporal cortex. Current Opinion in Neurobiology 15: 145–153. 15831395 10.1016/j.conb.2005.03.011

[29] James TW, Stevenson RA, Kim S, VanDerKlok RM, James KH (2011) Shape from sound: Evidence for a shape operator in the lateral occipital cortex. Neuropsychologia 49: 1807–1815. 21397616 10.1016/j.neuropsychologia.2011.03.004 PMC3100397

[30] Murray SO, Wojciulik E (2004) Attention increases neural selectivity in the human lateral occipital complex. Nat Neurosci 7: 70–74. 14647291 10.1038/nn1161

[31] Kim JG, Biederman I, Lescroart MD, Hayworth KJ (2009) Adaptation to objects in the lateral occipital complex (LOC): Shape or semantics? Vision Research 49: 2297–2305. 19577590 10.1016/j.visres.2009.06.020

[32] Ullman S (1995) Sequence Seeking and Counter Streams – a Computational Model for Bidirectional Information-Flow in the Visual-Cortex. Cerebral Cortex 5: 1–11. 7719126 10.1093/cercor/5.1.1

[33] Rao RP, Ballard DH (1999) Predictive coding in the visual cortex: a functional interpretation of some extra-classical receptive-field effects. Nat Neurosci 2: 79–87. 10195184 10.1038/4580

[34] Logothetis NK (2008) What we can do and what we cannot do with fMRI. Nature 453: 869–878. 18548064 10.1038/nature06976

[35] Altschuler TS, Molholm S, Russo NN, Snyder AC, Brandwein AB, et al (2012) Early electrophysiological indices of illusory contour processing within the lateral occipital complex are virtually impervious to manipulations of illusion strength. Neuroimage 59: 4074–4085. 22037001 10.1016/j.neuroimage.2011.10.051 PMC3288789

[36] Fiebelkorn IC, Foxe JJ, Schwartz TH, Molholm S (2010) Staying within the lines: the formation of visuospatial boundaries influences multisensory feature integration. Eur J Neurosci 31: 1737–1743. 20584177 10.1111/j.1460-9568.2010.07196.x

[37] Shpaner M, Murray MM, Foxe JJ (2009) Early processing in the human lateral occipital complex is highly responsive to illusory contours but not to salient regions. Eur J Neurosci 30: 2018–2028. 19895562 10.1111/j.1460-9568.2009.06981.x PMC3224794

[38] Lee TS, Mumford D (2003) Hierarchical Bayesian inference in the visual cortex. J Opt Soc Am A Opt Image Sci Vis 20: 1434–1448. 12868647 10.1364/josaa.20.001434

[39] Rao RPN (2005) Bayesian inference and attentional modulation in the visual cortex. Neuroreport 16: 1843–1848. 16237339 10.1097/01.wnr.0000183900.92901.fc

[40] Glover GH, Li TQ, Ress D (2000) Image-based method for retrospective correction of physiological motion effects in fMRI: RETROICOR. Magn Reson Med 44: 162–167. 10893535 10.1002/1522-2594(200007)44:1<162::aid-mrm23>3.0.co;2-e

[41] Bullmore ET, Long C, Suckling J, Fadili J, Calvert G, et al (2001) Colored noise and computational inference in neurophysiological (fMRI) time series analysis: Resampling methods in time and wavelet domains. Human Brain Mapping 12: 61–78. 11169871 10.1002/1097-0193(200102)12:2<61::AID-HBM1004>3.0.CO;2-W PMC6871881

[42] Purdon PL, Weisskoff RM (1998) Effect of temporal autocorrelation due to physiological noise and stimulus paradigm on voxel-level false-positive rates in fMRI. Human Brain Mapping 6: 239–249. 9704263 10.1002/(SICI)1097-0193(1998)6:4<239::AID-HBM4>3.0.CO;2-4 PMC6873371

[43] Birn RM (2012) The role of physiological noise in resting-state functional connectivity. Neuroimage 62: 864–870. 22245341 10.1016/j.neuroimage.2012.01.016 PMC13374118

[44] Cichy RM, Heinzle J, Haynes JD (2012) Imagery and perception share cortical representations of content and location. Cerebral Cortex 22: 372–380. 21666128 10.1093/cercor/bhr106

[45] Reddy L, Tsuchiya N, Serre T (2010) Reading the mind's eye: Decoding category information during mental imagery. Neuroimage 50: 818–825. 20004247 10.1016/j.neuroimage.2009.11.084 PMC2823980

[46] Stokes M, Thompson R, Cusack R, Duncan J (2009) Top-down activation of shape-specific population codes in visual cortex during mental imagery. J Neurosci 29: 1565–1572. 19193903 10.1523/JNEUROSCI.4657-08.2009 PMC6666065

[47] MacEvoy SP, Epstein RA (2011) Constructing scenes from objects in human occipitotemporal cortex. Nat Neurosci 14: 1323–1329. 21892156 10.1038/nn.2903 PMC6815103

[48] Larsson J, Heeger DJ (2006) Two retinotopic visual areas in human lateral occipital cortex. J Neurosci 26: 13128–13142. 17182764 10.1523/JNEUROSCI.1657-06.2006 PMC1904390

[49] Haushofer J, Livingstone MS, Kanwisher N (2008) Multivariate patterns in object-selective cortex dissociate perceptual and physical shape similarity. PLoS Biol 6: e187. 18666833 10.1371/journal.pbio.0060187 PMC2486311

