# Supplementary material for: Neural Associations of the Early Retinotopic Cortex with the Lateral Occipital Complex during Visual Perception
Source: PLoS One. 2014 Sep 24;9(9):e108557. doi: 10.1371/journal.pone.0108557 (PMC4177215; doi:10.1371/journal.pone.0108557)
Supplement: Text S1 — Supplementary Materials. (DOC) [file pone.0108557.s001.doc]

**Supplementary Materials**

**Performance of LOC patterns ‘predict’ V1 patterns using resting-state fMRI data**

We acquired two runs of resting-state fMRI (RS-fMRI) data from two other subjects, and used one run as the training data, and the other run as the validation data. The RS-fMRI data were acquired using a T2*-weighted gradient-echo echo-planar imaging (EPI) sequence with the following parameters: TR = 2000 ms, TE = 30ms, Flip angle = 90°, matrix = 64 × 64, FOV = 224 × 224 mm^2^, thickness/gap = 3.5/0.7 mm, 33 axial slices covering the whole brain. Each run consisted of 240 volumes. According to previous studies , we preprocessed the RS-fMRI data by using the following procedures: removing the first 10 volumes, slice timing, realignment to the first volume, spatial normalization to standard MNI space, band-pass filtering of 0.01 - 0.08 Hz, and regressing out the covariates (the six head motion parameters, and the white matter, CSF and global mean signals). Then, we localized the V1 according to the anatomical template , the left LOC with a sphere of 10 mm radius centered at [-39,-78,-9], and right LOC at [48,-75,0] . After defining the ROIs, we extracted the multiple-voxel patterns in V1 and LOC. Finally, using the same voxel-based encoding model (see the description in the main manuscript), we estimated the performance of LOC patterns to ‘predict’ V1 patterns when subjects were in resting-state. We found that the accuracy was just 0.9% for one subject, and 0% for the other subject, which is near to the chance level 0.4% (1/230).

We re-analyzed the resting-state fMRI data and investigated the influence of preprocessing steps (i.e., regression of head motion, global signal, and the white matter and CSF signals) on the final results. We found that the results were the same whether we regressed out these covariates or not, that is, the accuracy of LOC ‘predict’ ERC was 0.4% for one subject, and 0% for the other subject.

Although previous studies suggested that the regression techniques can reduce the physiological and motion artifacts and have been widely used in fMRI studies, especially in resting-state functional connectivity studies (see review in ), it is still controversial on the effect of the regression on fMRI data. For example, it has been shown that global signal regression can induce anti-correlations with the seed region being investigated , but the global signal may contain part of signal of interest and regressing out this global signal may therefore remove some of useful information. It is currently unclear to what extent that these nuisance regression can account for all of physiological and motion related noise, or whether these approaches are complementary to model-based methods . Thus, the future studies need continue to develop advanced correction methods to evaluate the physiological and motion effects.

**Fig. S1. The inverse modulations to the sub-areas of early retinotopic cortex between stimulus VBEM and LOC AVBEM for subject S2.**


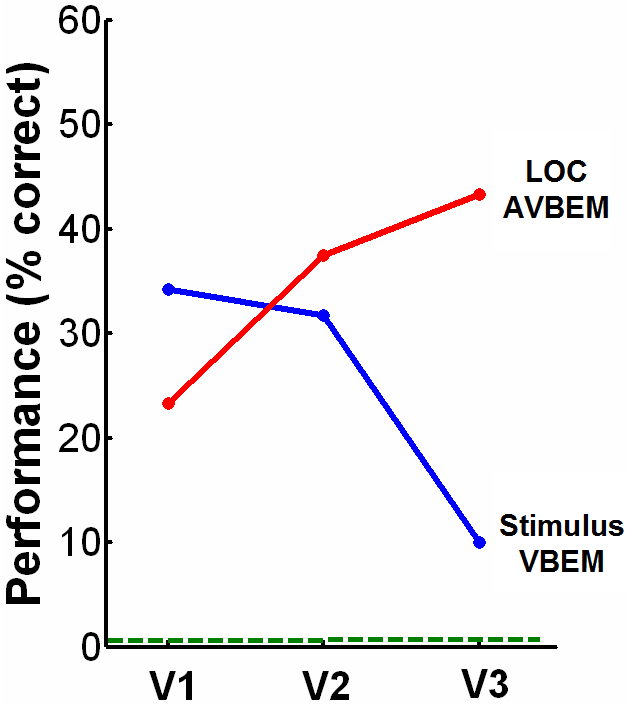


**References**

1. Chen G, Chen G, Xie C, Ward BD, Li W, et al. (2012) A method to determine the necessity for global signal regression in resting-state fMRI studies. Magn Reson Med 68: 1828-1835.

2. van den Heuvel MP, Hulshoff Pol HE (2010) Exploring the brain network: a review on resting-state fMRI functional connectivity. Eur Neuropsychopharmacol 20: 519-534.

3. Eickhoff SB, Stephan KE, Mohlberg H, Grefkes C, Fink GR, et al. (2005) A new SPM toolbox for combining probabilistic cytoarchitectonic maps and functional imaging data. Neuroimage 25: 1325-1335.

4. Grill-Spector K, Kourtzi Z, Kanwisher N (2001) The lateral occipital complex and its role in object recognition. Vision Res 41: 1409-1422.

5. Moore C, Engel SA (2001) Neural response to perception of volume in the lateral occipital complex. Neuron 29: 277-286.

6. Birn RM (2012) The role of physiological noise in resting-state functional connectivity. Neuroimage 62: 864-870.

7. Jones TB, Bandettini PA, Birn RM (2008) Integration of motion correction and physiological noise regression in fMRI. Neuroimage 42: 582-590.

8. Murphy K, Birn RM, Handwerker DA, Jones TB, Bandettini PA (2009) The impact of global signal regression on resting state correlations: are anti-correlated networks introduced? Neuroimage 44: 893-905.

9. Weissenbacher A, Kasess C, Gerstl F, Lanzenberger R, Moser E, et al. (2009) Correlations and anticorrelations in resting-state functional connectivity MRI: a quantitative comparison of preprocessing strategies. Neuroimage 47: 1408-1416.
